# Evidence synthesis of Chinese medicine for monkeypox: Suggestions from other contagious pox-like viral diseases

**DOI:** 10.3389/fphar.2023.1121580

**Published:** 2023-03-13

**Authors:** Hong-guo Rong, Xiao-wen Zhang, Mei Han, Xin Sun, Xiao-dan Wu, Xiao-zhen Lai, Chen Shen, Wei-jie Yu, Hai Fang, Yu-tong Fei, Jian-ping Liu

**Affiliations:** ^1^ Center for Evidence-Based Chinese Medicine, Beijing University of Chinese Medicine, Beijing, China; ^2^ Institute for Excellence in Evidence-Based Chinese Medicine, Beijing University of Chinese Medicine, Beijing, China; ^3^ School of Traditional Chinese Medicine, Beijing University of Chinese Medicine, Beijing, China; ^4^ China Center for Health Development Studies, Peking University, Beijing, China; ^5^ The National Research Center in Complementary and Alternative Medicine (NAFKAM), Department of Community Medicine, Faculty of Health Science, UiT The Arctic University of Norway, Tromsø, Norway

**Keywords:** monkeypox, smallpox, measles, varicella, rubella, Chinese medicine, clinical studies

## Abstract

**Background:** Monkeypox, a zoonotic disease caused by an *Orthopoxvirus*, presents an etiology similar to smallpox in humans. Currently, there are no licensed treatments for human monkeypox, so clear and urgent research on its prophylaxis and treatment is needed.

**Objective:** The purpose of this study was to explore the evidence of Chinese medicine for contagious pox-like viral diseases and provide suggestions for the multi-country outbreak management of monkeypox.

**Methods:** The review was registered on INPLASY (INPLASY202270013). Ancient classics in China and clinical trials involving randomized controlled trials , non-RCTs, and comparative observational studies of CM on the prevention and treatment of monkeypox, smallpox, measles, varicella, and rubella were retrieved from the Chinese Medical Code (fifth edition), Database of China Ancient Medicine, PubMed, the Cochrane Library, China National Knowledge Infrastructure, Chongqing VIP, Wanfang, Google Scholar, International Clinical Trial Registry Platform, and Chinese Clinical Trial Registry until 6 July 2022. Both quantitative and qualitative methods were applied to present the data collected.

**Results:** The use of CM to control contagious pox-like viral diseases was traced back to ancient Chinese practice cited in *Huangdi’s Internal Classic*, where the pathogen was recorded nearly two thousand years back. There were 85 articles (36 RCTs, eight non-RCTs, one cohort study, and 40 case series) that met the inclusion criteria, of which 39 studies were for measles, 38 for varicella, and eight for rubella. Compared with Western medicine for contagious pox-like viral diseases, CM combined with Western medicine showed significant improvements in fever clearance time (mean difference, −1.42 days; 95% CI, −1.89 to −0.95; 10 RCTs), rash/pox extinction time (MD, −1.71 days; 95% CI, −2.65 to −0.76; six RCTs), and rash/pox scab time (MD, −1.57 days; 95% CI, −1.94 to −1.19; five RCTs). When compared with Western medicine, CM alone could reduce the time of rash/pox extinction and fever clearance. Chinese herbal formulas, including modified Yinqiao powder, modified Xijiao Dihaung decoction, modified Qingjie Toubiao decoction, and modified Shengma Gegen decoction, were frequently applied to treat pox-like viral diseases and also showed significant effects in shortening the time of fever clearance, rash/pox extinction, and rash/pox scabs. Compared with Western medicine (placental globulin) or no intervention, eight non-randomized trials and observational studies on the prevention of contagious pox-like viral diseases showed a significant preventive effect of Leiji powder among high-risk populations.

**Conclusion:** Based on historical records and clinical studies of CM in managing contagious pox-like viral diseases, some botanical drugs could be an alternative approach for treating and preventing human monkeypox. Prospective, rigorous clinical trials are urgently needed to confirm the potential preventive and treatment effect of Chinese herbal formulas.

**Systematic Review Registration:** [https://inplasy.com/], identifier [INPLASY202270013].

## Introduction

An outbreak of monkeypox is occurring in multiple countries across the world, showing the characteristics of wide coverage, fast transmission, unclear epidemic focus, mainly human transmission, and accelerated community transmission and virus variation (Guarner et al., 2022). The multi-country monkeypox epidemic has become a serious public health emergency and requires a coordinated international response (Wenham and Eccleston-Turner, 2022). The World Health Organization (WHO) announced on 23 July 2022 that monkeypox outbreaks in several countries and regions constituted a public health emergency of international concern (PHEIC).

The most common symptoms of monkeypox identified during the 2022 outbreak include fever, headache, muscle aches, back pain, low energy, and swollen lymph nodes, followed or accompanied by the development of a rash that may last for 2 to 3 weeks (Patel et al., 2022). Caused by the monkeypox virus, monkeypox was recognized as the most significant *Orthopoxvirus* infection since the eradication of smallpox (Adler et al., 2022). Since the epidemic began to spread in early May 2022, the WHO has attached great importance to this special situation, rapidly releasing public health and clinical guidelines, engaging with communities actively, and convening scientists and researchers to accelerate the study of new diagnostic methods, vaccines, and treatments for monkeypox. At present, there are three kinds of vaccines against monkeypox, but the current supply is limited and there is a lack of data on the effectiveness of the new vaccine against monkeypox (Kumar et al., 2022; Looi, 2022). It is urgent to find new approaches to prevent and treat multi-country monkeypox outbreaks.

At the time of writing, there are no specific treatments for monkeypox patients as per the WHO and US Center for Disease Control and Prevention, and the treatment is mainly for the symptoms (Rizk et al., 2022). Monkeypox presents with fever, an extensive characteristic rash, and usually swollen lymph nodes (Adler et al., 2022; Orviz et al., 2022; Tarín-Vicente et al., 2022). It is important to distinguish monkeypox from other contagious pox-like viral diseases such as varicella, measles, bacterial skin infections, scabies, syphilis, and medication-associated allergies. However, we can explore ways to prevent and treat monkeypox from human experience in fighting against other contagious pox-like viral diseases. Throughout the 3000-year history of China, Chinese medicine (CM) has been used as the routine treatment regime for acute infectious diseases (Wu et al., 2021). For example, when severe acute respiratory syndrome (SARS) was the most serious infectious disease outbreak in China in 2003, Chinese herbal formulas played a significant role in the prevention and treatment of SARS (Liu et al., 2004; Liu et al., 2012). In 2022, facing the outbreak of monkeypox around the world, the National Health Commission of China issued a CM treatment program in the *Guidelines for the Diagnosis and Treatment of Monkeypox (2022 Version)* (Interpretation of guidelines for diagnosis, 2022), which included six Chinese herbal formulas based on different clinical symptoms.

At present, the multi-country outbreak of monkeypox has drawn worldwide attention, and it is necessary to find appropriate solutions as soon as possible. Therefore, we conducted this review to identify the historical and human research evidence and critically review the potential for CM to prevent and treat monkeypox.

## Methods

### Data sources

The protocol of this review has been registered on INPLASY (INPLASY202270013). Two types of data in the current study were searched, including historical classics records and human research evidence. Historical classics records are records on the prevention and treatment of contagious pox-like viral diseases in ancient CM books, including history, treatment principles, medicines, and the application of CM to prevent or treat contagious pox-like viral diseases. Human research studies are studies to investigate the preventive and therapeutic effects of CM on monkeypox, smallpox, measles, varicella, and rubella were included. The inclusion criteria were as follows: 1) study design: clinical trials, clinical-controlled trials, cohort studies, cross-sectional studies, case-control studies, case series, and clinical observations; 2) population: high-risk populations exposed to monkeypox, smallpox, measles, varicella, and rubella; 3) intervention: CM should be used alone or combined with conventional treatment or healthcare; 4) control: conventional treatment, healthcare, placebo, or blank control; and 5) outcome: outcomes included all-cause mortality, pathogen clearance rate or time, and improvement of overall symptoms, including but not limited to fever, frequency, and severity of pox.

### Literature search

The retrieval strategies of the aforementioned two types of data were different. The Chinese Medical Code (fifth edition) and Database of China Ancient Medicine were taken as the retrieval source of ancient literature. The list of literature retrieved was determined after discussions among all authors. PubMed, the Cochrane Library, China National Knowledge Infrastructure, Chongqing VIP, Wanfang, Google Scholar, International Clinical Trial Registry Platform, and Chinese Clinical Trial Registry were taken as the retrieval source of modern literature. The search date was up to 6 July 2022.

Taking PubMed as an example, the retrieval terms were listed as follows:

#1 Monkeypox OR monkey pox OR monkeypox virus OR variole du singe OR variole simienne OR smallpox OR variola OR measles OR measles virus OR varicella OR chickenpox OR varicellovirus OR rubella OR rubeola OR Rubella virus OR german measles [title, abstract, keywords]
#2 traditional Chinese medicine OR Chinese medicine OR traditional medicine OR Chinese herbal medicine OR ethnological medicine OR Chinese materia medica OR Chinese patent medicine [title, abstract, keywords]
#3 trial OR participants OR patients OR randomized controlled trial OR random OR controlled clinical trial OR placebo OR cohort study OR case-control study OR epidemiology OR cross-sectional study OR descriptional study OR population-based OR clinical observation [title, abstract, keywords].

### Data extraction and analysis

For CM classics, we summarized the text related to pox-like viral diseases with a narrative description. For clinical trials, we designed a data extraction form for study characteristics and outcome data, which we piloted on at least one study in the review. The study characteristics were extracted by two authors independently, including the characteristics of the population (age), the type of therapies, treatment methods and courses, reported outcomes, and adverse events/reactions. According to the Convention on the Rights of the Child, our study defines children as “every human being below the age of eighteen years.” Any disagreement was solved by a third author to reach a consensus.

The following data were extracted and analyzed: source of evidence, time of publication or release, author, setting, the basis for the formulation of CM prophylaxis and treatment strategy, the composition of CM prescription, target disease, course of prevention, effect, and adverse reaction. The data were qualitatively described and presented, and if possible, quantitative or descriptive statistics were conducted.

When the data were available for pooling, meta-analysis would be conducted by RevMan 5.3 software. Considering the trials usually applied different Chinese herbal formulas, we have to pool the data together to explore the overall effectiveness of CM, so a random-effects model was applied for all the pooled data. The mean difference (MD) was used for continuous data, and relative risk (RR) for dichotomous data, both with 95% confidence intervals (CI).

## Results

### General description of contagious pox-like viral diseases in ancient CM classics

**FIGURE 1:**
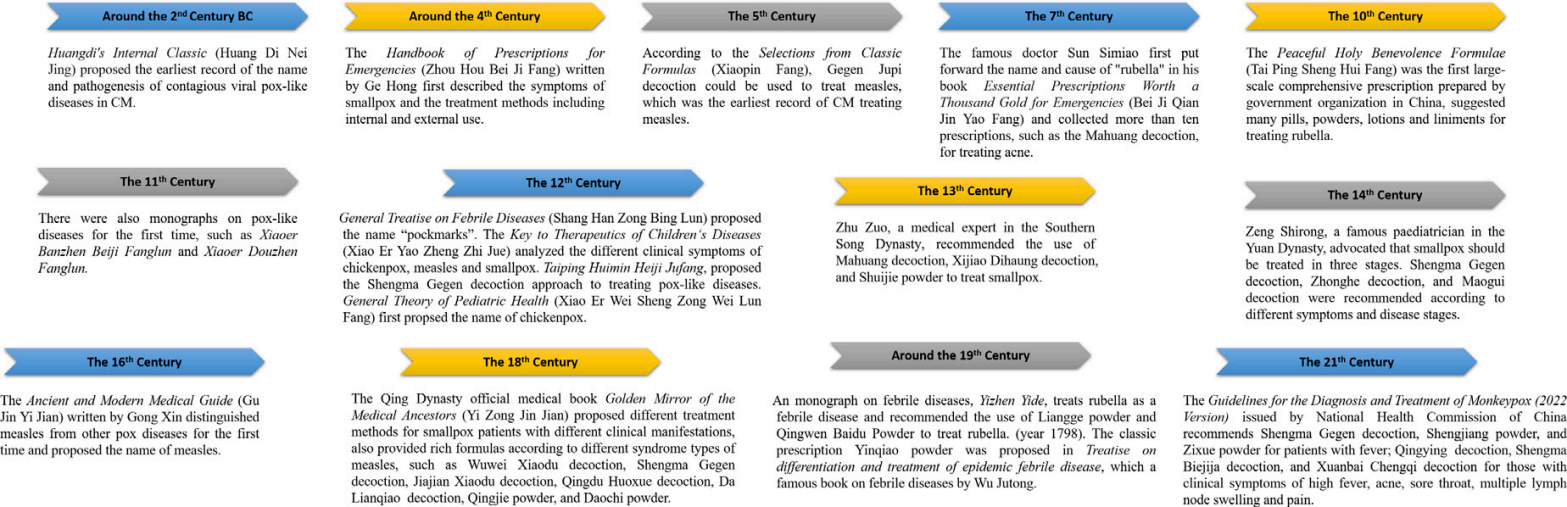
Brief history of CM for prevention and treatment of contagious pox-like viral diseases.

Historically, CM has rich experience in treating contagious pox-like viral diseases (Figure 1). The first record of contagious pox-like viral diseases dated back to *Huangdi’s Internal Classic*, written about 2,000 years ago. *Huangdi’s Internal Classic* reported that surplus qi of lesser yin led to skin impediment and urticaria, which was considered to be the earliest record of the name and pathogenesis of contagious pox-like viral diseases. The *Suwen Great Theory on Normality of Six-Qi*, part of *Huangdi’s Internal Classic*, reported that the occurrence of these diseases was marked by body fever, vomiting, cholera, carbuncles, soreness, and ulceration. These theories had a profound impact on the treatment of contagious pox-like viral diseases.

Monkeypox virus and smallpox virus belonged to the genus *Orthopoxvirus* of Poxviridae. The *Handbook of Prescriptions for Emergencies* written by Ge Hong in the Eastern Jin Dynasty first described the symptoms of smallpox and the treatment methods including internal and external use. A famous pediatrician in the Northern Song Dynasty, Qian Yi wrote the *Key to Therapeutics of Children’s Diseases* and analyzed the different clinical symptoms of varicella, measles, and smallpox. The name varicella was first proposed in *General Theory of Pediatric Health* in the Southern Song Dynasty, as, “the skin of the sore is very thin, like blisters, and it dries quickly when punctured, which is called varicella.” Medical books before the Ming Dynasty, such as *Essential Prescriptions Worth a Thousand Gold for Emergencies*, *Medical Secrets of an Official*, and *General Treatise on Febrile Diseases*, had a preliminary understanding of the clinical manifestations of measles. *General Treatise on Febrile Diseases* also proposed the name “pockmarks” and distinguished it from smallpox. *Ancient and Modern Medical Guide* written by Gong Xin, a famous doctor in the Ming Dynasty, distinguished measles from other pox diseases for the first time, proposed the name measles, and recorded the diagnosis and treatment of measles in detail. The famous doctor Sun Simiao (541–682 AD) first put forward the name and cause of “rubella” in his book *Essential Prescriptions Worth a Thousand Gold for Emergencies*, which said that “when the wind pathogen invades the skin, the deficiency itches then changed into rubella, itching, and sores.”

### Chinese herbal formulas for the prevention and treatment of contagious pox-like viral diseases in ancient CM classics

CM has been used to treat smallpox since ancient times, and there were various methods. The famous doctor Ge Hong first proposed that smallpox could be treated by internal and external use of CM in his book *Handbook of Prescriptions for Emergencies*. It could be taken orally with honey-fried *Actaea cimicifuga* L. and scrubbed externally with boiled *A. cimicifuga* L. *Essential Prescriptions Worth a Thousand Gold for Emergencies* was a classic work of CM written by Sun Simiao, a Chinese physician titled China’s King of Medicine in the Tang Dynasty. This book collected more than 10 prescriptions for treating acne. Most of the medicines used in these prescriptions were mainly cool feature medicines, such as *Coptis chinensis* Franch., *Indigofera suffruticosa* Mill., Cornu Bubali, *Rheum officinale* Baill., and *Mangifera indica* L. In the Song Dynasty, the treatment methods for smallpox became more abundant. A medical expert Zhu Zuo recommended the Mahuang decoction, Xijiao Dihaung decoction, and Shuijie powder to treat smallpox. In the Song Dynasty, there were also monographs on pox-like diseases, such as *Xiaoer Banzhen Beiji Fanglun* and *Xiaoer Douzhen Fanglun*, which discussed the etiology, syndrome differentiation, treatment principles, and presentations of children’s pox and rash and provided many treatment approaches in this period. Thereafter, the prevention and treatment of smallpox with CM have been practiced in depth, improving its treatment methods. Zeng Shirong, a famous pediatrician in the Yuan Dynasty, advocated that smallpox should be treated in three stages. When the pox rash did not occur, Shengma Gegen decoction plus *Ephedra sinica* Stapf could be used to promote the eruption. If the pox rash has occurred, Zhonghe decoction could be used to smooth the appearance and interior. If acne was easy to grow, Maogui decoction could be used to replenish qi, promote blood circulation, and detoxify. If the rash did not scab for a long time, Shengma Gegen decoction plus *E. sinica* Stapf could be used to promote scab formation. After a rash scab, the Shengma Gegen decoction could be used for detoxification. The numbers of monographs on pox and rash appeared in the Ming and Qing dynasties. The theory was mature, and the description was complete. For example, the Qing Dynasty’s official medical book *Golden Mirror of the Medical Ancestors* proposed different treatment methods for smallpox patients with different clinical manifestations: Wuwei Xiaodu decoction could be used when the pox started to increase. If there was fever on the body surface but no sweat, Shengma Gegen decoction was recommended. If the viscera were hot and sweaty, Jiajian Xiaodu decoction was recommended. If herpes forms and the fever did not subside, Qingdu Huoxue decoction was recommended. If fever occurred after acne, Da Lianqiao decoction was recommended. If convulsions occurred before the appearance of the rash, it was recommended to use Qingjie powder. If still had convulsive symptoms after the rash, it was recommended to use Daochi powder.

For the ancient CM treatment of measles, the basic therapeutic principles and methods for measles were clearing heat and promoting eruption. The earliest record of CM for measles can be traced back to the *Selections from Classic Formulae* published in the Eastern Jin Dynasty. This book suggested that Gegen Jupi decoction could be used to treat measles. In the monograph on the pediatrics of CM in the Southern Song Dynasty, *Xiaoer Weisheng Zongwei Lunfang* opened a special column on disease diagnosis and treatment of “acne and rash theory” and recommended a variety of formulas to treat acne and rash diseases. For example, Leiji powder has an effective effect on the treatment of children’s acne and rash characterized by irritability and thirst, soreness in the mouth and tongue, and obstruction of urine. Another famous medical book in the same period, *Taiping Huimin Heiji Jufang* (the first proprietary medicine standard compiled by the government in the world), established the Shengma Gegen decoction approach to treating pox-like diseases, which was widely used. The *Ancient and Modern Medical Guide* in the Ming Dynasty put forward the viewpoint of “warming and tonifying” to treat measles according to syndrome differentiation. The formulas include modified Shengma Gegen decoction and modified Siwu decoction. In the Qing Dynasty, there were more CM prescriptions for measles. In an important work on febrile diseases, the classic prescription Yinqiao powder was proposed in *Treatise on Differentiation and Treatment of Epidemic Febrile Disease*, a famous book about febrile diseases. As the basis of summing up predecessors’ experience, *Golden Mirror of the Medical Ancestors* in the Qing Dynasty provided rich formulas according to different syndrome types of measles, such as Shengma Gegen decoction, Maxing Shigan decoction, Sanhaung Shigao decoction, and Siwei decoction.

The methods of CM treatment for varicella were clearing heat, resolving dampness, and removing toxins. According to the different syndromes of varicella, the methods of releasing exterior and clearing interior, clearing qi aspect and cooling nutrient aspect, eliminating dampness, and removing toxins were, respectively, used. In the Song Dynasty, although *Xiaoer Weisheng Zongwei Lunfang* first proposed the name varicella, it is still considered a combination of pox-like viral diseases in terms of treatment. It mentioned a variety of treatments, such as Shengma Gegen decoction, Leiji powder, and Baidu powder. *Baoying Cuoyao* in the Ming Dynasty contains important medical treatises from the Han to the Tang, Song, Jin, and Yuan dynasties, and down to the early Ming Dynasty, reflecting the continuation and development of the history of pediatrics theory in CM. *Baoying Cuoyao* suggested that for patients with the pathogenic qi light shallow, mainly exterior syndrome, Shengma Gegen decoction should be selected. In the Qing Dynasty, CM practitioners added the prescriptions of predecessors and skillfully applied them to treat varicella. For example, the *Golden Mirror of the Medical Ancestors* mostly adopted the formula of strengthening the body and resolving the exterior, clearing away heat and diuresis to treat varicella.

The methods of CM treatment for rubella were mainly based on clearing heat to release exterior, cooling blood and promoting eruption, and harmonizing nutrient and defensive aspects. Sun Simiao recorded the Mahuang decoction to treat rubella in his book *Essential Prescriptions Worth a Thousand Gold for Emergencies*. *Peaceful Holy Benevolence Formulae* was the first large-scale comprehensive prescription prepared by a government organization in China, which collated the previous experience in treating diseases, and later generations’ empirical prescriptions, time prescriptions, exotic drugs, *etc. Treatise on differentiation and treatment of epidemic febrile disease*, a treatise on febrile diseases in the Qing Dynasty, pointed out for the first time that although the eruption was not difficult to treat, the treatment was urgent and the time limit was only 3 days, and said that the exterior should be released with pungent cool medicine first and then recovered using sweet cool medicine. In another monograph on febrile diseases, *Yizhen Yide* recommended the Liangge powder and Qingwen Baidu powder to treat rubella.

### Clinical studies’ search results

**FIGURE 2:**
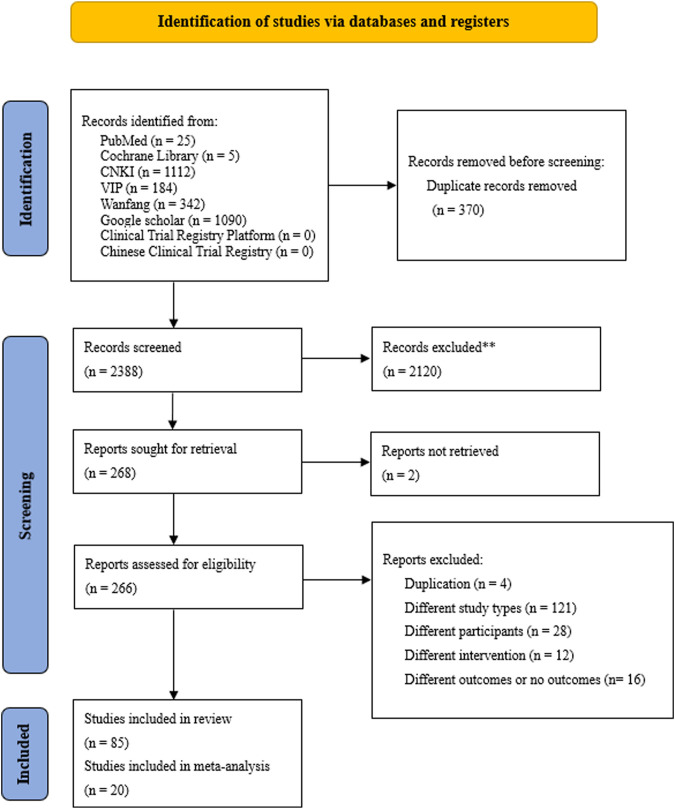
Flow chart of the study selection process.

Across searches, 2,758 studies were retrieved. After deduplication, 2,388 records remained. After screening titles and abstracts for relevance, 268 records remained. Two reports were not retrieved. After reading the full text, 76 records remained. After reviewing reference lists, eight studies were added. In total, 85 articles met the inclusion criteria for the current study objective. Of the 85 studies, 36 were randomized controlled trials (RCTs), eight non-RCTs, one cohort study, and 40 case series. There were 39 pieces of literature on measles, 38 on varicella, eight on rubella, and none on monkeypox or smallpox. The flow of articles through identification to final inclusion is represented in Figure 2.

### Evidence of Chinese herbal formulas for prevention and treatment of measles

Thirty-nine studies were identified, including 11 RCTs, seven non-RCTs, one cohort study, and 20 case series. Table 1 presents the studies on the prevention of measles by CM, including one RCT, four non-RCTs, and four case series. The Chinese herbal formulas of Leiji powder were widely used in the prevention of measles. The population comprised children who were directly or indirectly in contact with confirmed measles cases, with ages ranging from 4 months to 15 years (three papers did not report the specific age of children). The total sample size was 15,566, with the largest one being 7,828. The course of taking Leiji powder for prevention was 3–30 days. The incidence rate of measles ranged from 1.7% to 71.7%. The results of one study (Chen, 1964) showed that the effect of using Leiji powder to prevent measles is not evident. Other studies have proved that Leiji powder could significantly reduce the incidence rate of measles. Even after the occurrence of measles, the use of Leiji powder could significantly improve its clinical symptoms.

**TABLE 1:** Characteristics of clinical trials on Leiji powder preventing measles.

Study ID	Study type	Sample size (case, T/C)	Average age (year)	Intervention	Control	Treatment course (d)	Follow-up (d)	Incidence number (n)	Incidence rate (%)
Chen 1964 (Chen, 1964)	RCT	T: 53	T: 6 months–15	Modified Leiji powder	Blank	14	60	T: 38	T: 71.7%
		C: 50	C: 6 months–15					C: 32	C: 64.0%
Zhang 1960 (Zhang, 1960)	Non-RCT	T1: 847	0–10	T1	Blank	12–15	27–28	T1: 11	T1: 1.3%
		T2: 259		*Ferula sinkiangensis* K.M.Shen				T2: 13	T2: 5.0%
		C: 205		T2: Leiji powder				C: 26	C: 31.0%
Jinzhou 1959 (Jinzhou city hospital, 1959)	Non-RCT	T1: 742	Not reported	T1: Fangma decoction	Placental globulin	T1: not reported	Not reported	T1: 208	T1: 28.0%
		T2: 3,460		T2: Guanzhong powder		T2: 3		T2: 692	T2: 20.0%
		T3: 1,595		T3: Goose egg		T3: 45		T3: 479	T3: 30.0%
		T4: 2031		T4: Leiji powder		T4: 7–10		T4: 349	T4: 17.2%
		C: not reported							C: 45%
Yan 1959 (Yan and Su, 1959)	Non-RCT	T: 75	Not reported	Leiji powder	Placental globulin and umbilical placenta powder	30	More than 1 month	41	T: 54.7%
		C: 35							C: 71.4%
Lu 1957 (Lu, 1957)	Non-RCT	T1: 261	6 months–12	T1: Zicaogen decoction	Blank	T1: 3	T1: 56	T1: 48	T1: 18.4%
		T2: 145		T2: Leiji powder		T2: 5	T2: 30	T2: 9	T2: 6.2%
		C: 60							C: 14.6%
Sheng 1957 (Lu, 1957)	Case series	1,086	4 months–10	Leiji powder	Not reported	25	21	18	1.7%
Zheng 1960 (Zheng, 1960)	Case series	4,468	Not reported	Leiji powder	Not reported	5	Not reported	648	14.5%
Qiang 1959 (Qian et al., 1959)	Case series	T1: 118	4 months–7	Leiji powder	Not reported	T1: 28	T1: 30	31	T1: 26.3%
		T2: 25				T2: 3	T2: 40		T2: 8.0%
		T3: 92				T3: 5	T3: 30		T3: 3.3%
Xinxiangg 1959 (Measles prevention and control group of Shengli road office of Xinxiang City, 1959)	Case series	164	4 months–7	Leiji powder	Not reported	7–10	15	3	1.8%

Notes: RCT, randomized controlled trial; T, treatment group; and C, control group.

Table 2 shows the basic characteristics of studies (10 RCTs, three non-RCTs, and one cohort study) on CM treatment for measles. Most of the included studies had clear diagnostic criteria of CM or Western medicine for measles (Supplementary Table S1). The participants of those studies were adults and children, the youngest of whom was 4 months old. The total sample size was 2,277, with the largest one being 899. The treatment therapeutic principles and methods of CM for measles are based on clearing heat, detoxifying, and penetrating rash (Supplementary Table S1). These included interventions comprise three self-made prescriptions and 14 classic Chinese herbal formulas. Modified Qingjie Toubiao decoction appeared most frequently in the classic prescriptions of CM (3/14), followed by modified Yinqiao powder, modified Yinqiao powder, and modified Maxing Shigan decoction (all 2/14). The specific components of the prescription are shown in Supplementary Table S1. The conventional anti-symptomatic treatment was generally used as a control. Five RCTs and one non-RCT reported the course of formulas ranging from 3 to 10 days. The main outcome measures were the total effective rate, fever clearance time, time of rash disappearance, and complication rate. Two studies reported the adverse events including nausea, vomiting, and bad appetite, while two studies reported that no adverse events occurred, and the others did not report on this aspect.

**TABLE 2:** Characteristics of included trials of Chinese herbal formulas for measles.

Study ID	Design type	Population	Average age (year)	Sample size (case, T/C)	Intervention	Control	Course (d)	Outcome	Adverse reaction
Xu 2020 (Xu, 2020)	RCT	Children	T: 5.28 ± 1.11	80 (40/40)	Modified Yinqiao powder	Conventional anti-symptomatic treatment	3–5	Total effective rate and complication rate	Not reported
			C: 5.42 ± 1.06						
Zhou 2019 (Zhou et al., 2019)	RCT	Children	T: 5 months–14	60 (30/30)	Self-made prescription	Conventional anti-symptomatic treatment	7	Total effective rate, time of rash disappearance, fever clearance time, duration of cough, hospitalization time, and complication rate	Not reported
			C: 4 months–13						
Tu 2016 (Tu et al., 2016)	RCT	Adult	T1: 35.79 ± 7.12	87 (30/29/28)	Modified Qingjie Toubiao decoction	Conventional anti-symptomatic treatment	Not reported	Total effective rate, fever clearance time, hospitalization time, duration of fever, and complication rate	Nausea and vomiting
			T2: 36.46 ± 10.53						
			C: 32.23 ± 7.38						
Li 2015 (Li and Lei, 2015)	RCT	Adult	32.21 ± 1.83	100 (50/50)	Self-made prescription	Conventional anti-symptomatic treatment	Not reported	Hospitalization time and total effective rate	Not reported
Liu 2013 (Liu and Liang, 2013)	RCT	Children	T: 7.35 ± 3.12	60 (30/30)	Self-made prescription	Combined traditional Chinese and Western medicine	Not reported	Hospitalization time and complication rate	Not reported
			C: 7.71 ± 2.54						
Zhang 2009 (Zhang et al., 2009)	RCT	Adult	T: 22.7 ± 15.6	62 (32/30)	Modified Huanglian Jiedu decoction and modified Xijiao Dihaung decoction	Conventional anti-symptomatic treatment	5	Total effective rate, scores of clinical symptoms and signs, normalization rate of irritative cough liver function normalization rate, hospitalization time, and medical cost	Not reported
			C: 23.5 ± 16.7						
Niu 2006 (Niu et al., 2006)	RCT	Children	T: 4.58 ± 1.41	100 (50/50)	Modified Qingjie Toubiao decoction	Conventional anti-symptomatic treatment	Not reported	Total effective rate, fever clearance time, rash onset time, and the disappearance time of pulmonary rales	Not reported
			C: 4.66 ± 1.50						
Zhang 2004 (Zhao et al., 2004)	RCT	Children/adult	Not reported	86 (44/42)	Modified Maxing Shigan decoction	Conventional anti-symptomatic treatment	4–10	Fever clearance time, time of rash disappearance, markedly effective, and complication rate	Not reported
Li 2000 (Li, 2000)	RCT	Children	T: 3.48 ± 0.31	102 (60/42)	Modified Xijiao Dihaung decoction	Conventional anti-symptomatic treatment	Not reported	Fever clearance time, rash onset time, course of disease, and total effective rate	Not reported
			C: 3.26 ± 0.54						
Wang 1995 (ang, 1995)	RCT	Children/adult	T: 1–25	142 (82/60)	Modified Qingjie Toubiao decoction and modified ephedra, apricot, gypsum, and licorice decoction	Conventional anti-symptomatic treatment	7–10	Cure rate	Not reported
			C: not reported						
Zhao 2021 (Zhao, 2021)	Non-RCT	Children	T: 3.58 ± 1.02	100 (50/50)	Modified Xuanbai Chengqi decoction	Routine general care	Not reported	Time of rash disappearance, fever clearance time, duration of cough, sleepiness time, total effective rate, and degree of satisfaction	Not reported
			C: 4.15 ± 1.01						
Ye 1962 (Ye et al., 1962)	Non-RCT	Children	Not reported	899 (818/81)	Modified Sangju decoction, modified Yinqiao powder, and modified Qianjin Weijing decoction	Conventional anti-symptomatic treatment	4.38	Fever clearance time, time of rash disappearance, cure time, and complication rate	Not reported
303 Hospital 1959 (Efficacy effect in 155 cases, 1959)	Non-RCT	Not reported	Not reported	192 (155/37)	Zicao Fuping decoction and Sanhaung Shigao decoction	Western medicine antibiotic treatment	Not reported	Rash onset time and complication	Not reported
Qi 2016 (Qi, 2016)	Cohort study	Adult	33	80 (40/40)	Modified Shengma Gegen decoction	Conventional anti-symptomatic treatment	Not reported	Duration of fever, WBC, CRP, and ALT, and complication	Bad appetite

Notes: RCT, randomized controlled trial; T, treatment group; C, control group; WBC, white blood cell; CRP, C-reactive protein; and ALT, alanine transaminase.

### Evidence of Chinese herbal formulas for varicella

Thirty-eight studies were identified, including 22 RCTs, one non-RCT, and 15 case series. All the studies were published in Chinese. The details of the characteristics of included trials RCTs and non-RCTs are presented in Table 3. More than half of the studies reported having clear diagnostic criteria of CM or Western medicine for varicella (Supplementary Table S2). The participants were children and adults, ranging from 4 months to 58 years old. The total sample size of the included clinical studies was 2,015. The therapeutic principles and methods for varicella were clearing heat and cooling blood and removing toxins and dampness (Supplementary Table S2). These included interventions comprising 15 classic Chinese herbal formulas, three self-made prescriptions, and one Chinese patent medicine (Lianhua Qingwen Capsules). Among these, modified Yinqiao powder was applied most frequently (7/15), followed by Xijiao Dihaung decoction (5/15). Shengma Gegen decoction, Baihu decoction, and modified Wuwei Jiedu decoction appeared three times. Modified Qingjie Toubiao decoction, Sanren decoction, and modified Liuyi powder appeared two times. The specific composition of the prescription is shown in Supplementary Table S1. The control group was generally treated with Western medicine. Eighteen RCTs reported the course of Chinese herbal formulas ranging from 3 to 10 days. The main outcome measures included the total effective rate, fever clearance time, time of rash disappearance, rash scab time, and index tested by laboratory serological diagnosis. Twelve clinical studies reported adverse events that occurred, including nausea, vomiting, and bad appetite. Six studies reported that no adverse events occurred, while the others did not report on this aspect.

**TABLE 3:** Characteristics of included trials of Chinese herbal formulas for varicella.

Study ID	Design type	Population	Average age (year)	Sample size (case, T/C)	Intervention	Control	Course (d)	Outcome	Adverse reaction
Huang 2022 (Huang, 2022)	RCT	Children	T: 7.19 ± 2.10	84 (42/42)	Huanglian Jiedu decoction, Xijiao Dihaung decoction, and modified Six-one powder	Western medicine antiviral therapy	5	Total effective rate, CM syndrome scores, CRP, TNF-α, and PCT, fever clearance time, time of rash disappearance, rash scab time	Diarrhea, thirst, nausea, vomiting, headache, and skin itching
			C: 6.54 ± 2.23						
Zhang 2021 (Zhang and Zhou, 2021)	RCT	Children	T: 5.70 ± 1.80	60 (30/30)	Self-made prescription	Western medicine antiviral therapy	5	Total effective rate, CM syndrome scores, fever clearance time, time of rash disappearance, antipruritic time, hospitalization time, and Hamilton anxiety scale	No
			C: 6.00 ± 2.13						
Din 2021 (Ding, 2021)	RCT	Children	T: 9.80 ± 3.04	60 (30/30)	Self-made prescription	Western medicine antiviral therapy	5	Total effective rate, fever clearance time, and rash scab time	Not reported
			C: 9.77 ± 3.09						
Guan 2020 (Guan, 2020)	RCT	Children	T: 4.5 ± 0.5	200 (100/100)	Yinqiao powder and Three-nut decoction	Western medicine antiviral therapy	7	Total effective rate, fever clearance time, and time of rash disappearance	Not reported
			C: 4.7 ± 0.7						
Tang 2019 (Tang et al., 2019)	RCT	Adult	T: 32.09 (26–43)	57 (28/29)	Lianhua Qingwen capsules	Western medicine antiviral therapy	7	Total effective rate, fever clearance time, and rash scab time	Fever, headache and gastrointestinal discomfort
			C: 33.05 (29–49)						
Gao 2016 (Gao et al., 2016)	RCT	Adult	T: 22.9 ± 2.3	100 (50/50)	Modified Yinqiao powder and modified six-one powder	Western medicine antiviral therapy	10	Total effective rate, fever clearance time, time of rash disappearance, and cure time	Not reported
			C: 23.2 ± 2.1						
Zhang 2016a (Zhang, 2016)	RCT	Children	T: 5.7 ± 2.1	80 (40/40)	Shengma Gegen decoction, White Tiger decoction, and Xijiao Dihaung decoction	Western medicine antiviral therapy	7	Total effective rate, fever clearance time, rash scab time, and hospitalization time	Nausea, vomiting, proteinuria, and mild stomach discomfort
			C: 5.4 ± 1.4						
Han 2016 (Han, 2016)	RCT	Children	T: 4.3 ± 1.4	86 (43/43)	Shengma Gegen decoction, Baihu decoction, and Xijiao Dihaung decoction	Western medicine antiviral therapy	Not reported	Time of rash disappearance, total effective rate, and hospitalization time	Nausea, vomiting, and proteinuria
			C: 4.1 ± 1.7						
Wang 2015 (Wang, 2015)	RCT	Children	T: 3–8	60 (30/30)	Modified Wuli Huichun Pills	Western medicine antiviral therapy	Not reported	Total effective rate, fever clearance time, antipruritic time, rash scab time, and cure time	Not reported
			C: 2–7						
Quan 2011 (Quan, 2011)	RCT	Children	T: 2–12	200 (100/100)	Kushen decoction	Western medicine antiviral therapy	5	Total effective rate	No
			C: 2–12						
Chen 2010 (Chen, 2010)	RCT	Children	T: 1–10	60 (30/30)	Modified Yinqiao powder	Western medicine antiviral therapy	7	Total effective rate	Not reported
			C: 1–10						
Zeng 2009 (Zeng et al., 2009)	RCT	Children	T: 2–15	86 (44/42)	Modified Yinqiao powder and modified Qingwen Baidu decoction	Western medicine antiviral therapy	5	Total effective rate	Not reported
			C: 2–15						
Zhu 2009 (Zhu et al., 2009)	RCT	Children	T: 9.32 ± 2.15	85 (43/42)	Modified Yinqiao powder and modified Three-nut decoction	Western medicine antiviral therapy	3	Total effective rate, fever clearance time, time of rash disappearance, and rash scab time	No
			C: 9.28 ± 2.24						
Zhao 2009 (Zhao, 2009)	RCT	Adult	T: 21.4 (19–24)	80 (40/40)	Modified Qingjie Toubiao decoction	Western medicine antiviral therapy	Not reported	Fever clearance time, rash scab time, IL-6, IL-8, and TNF-α	No
			C: 21.6 (19–24)						
Zhao 2007 (Zhao, 2007)	RCT	Children	T: 7.55 ± 2.41	76 (42/34)	Modified Qingjie Toubiao decoction and modified Wuwei Xiaodu decoction	Western medicine antiviral therapy	5	Total effective rate, fever clearance time, antipruritic time, and rash scab time	Shivering, headache, nausea, vomiting, and bad appetite
			C: 7.25 ± 2.49						
Chen 2006 (Chen, 2006)	RCT	Adult	T: 18–58	86 (46/40)	Modified Youlong decoction	Western medicine antiviral therapy	7	Total effective rate	No
			C: 18–58						
Zhang 2006 (Zhang and Tao, 2006)	RCT	Children	T: 4.56 ± 1.28	48 (24/24)	Modified Yinqiao powder, Wuwei Xiaodu decoction, and Xijiao Dihaung decoction	Western medicine antiviral therapy	3	Total effective rate	Not reported
			C: 5.15 ± 1.42						
Yang 2003 (Yang, 2003)	RCT	Children	T: 3–7	114 (60/54)	Modified Yinqiao powder	Western medicine antiviral therapy	3	Total effective rate, fever clearance time, antipruritic time, time of rash disappearance, and rash scab time	Not reported
			C: 3–7						
Li 2002 (Li and Zhang, 2002)	RCT	Children	T: 9 months–12	68 (38/30)	Modified Wuwei Xiaodu decoction	Western medicine antiviral therapy	5	Total effective rate	No
			C: 1–10						
Li 1999 (Li, 1999)	RCT	Children	5.3 (3–14)	77 (45/32)	Modified *Coix* and bamboo leaf powder	Western medicine antiviral therapy	3.1	Total effective rate, fever clearance time, and rash scab time	Not reported
Wan 1996 (Wan and Ye, 1996)	RCT	Children	7 months–13	120 (60/60)	Modified Qingwei powder	Western medicine antiviral therapy	Not reported	Total effective rate	Not reported
Lin 1994 (Lin, 1994)	RCT	Children	T: 7 (4 months–13)	48 (25/23)	Self-made prescription	Western medicine antiviral therapy	6	Total effective rate, and rash scab time	Not reported
			C: 6.5 (6 months–11)						
Ma 2017 (Ma, 2017)	Non-RCT	Children	7 (3–11)	80 (40/40)	Shengma Gegen decoction, Baihu decoction, and Xijiao Dihaung decoction	Western medicine antiviral therapy	Not reported	Total effective rate	Adverse event rate

Notes: RCT, randomized controlled trial; T, treatment group; C, control group; CM, Chinese medicine; CRP, C-reactive protein; TNF-α, tumor necrosis factor-α; PCT, procalcitonin; IL-6; interleukin-6; and IL-8, interleukin-8.

### Evidence of Chinese herbal formulas for rubella

For Chinese herbal formulas for rubella, three RCTs and five case series were identified. The characteristics of included RCTs are presented in Table 4. The subjects were children or adults with confirmed rubella. The total sample size of the included clinical studies was 255, with the average age ranging from 1 to 27.93 years old. The tested interventions included modified Yinqiao powder, Xijiao Dihaung decoction, and Chinese patent medicines (Huanglan Granule). The control measures mainly included Western medicine antiviral treatment (ribavirin), classic Chinese herbal formulas, and self-made prescription. Two studies reported that the course of formulas lasted for 3–7 days and 20 days, respectively. The main outcome measures included fever clearance time, the time of rash disappearance, and RuV-IgM. One study reported that no adverse events occurred, while the others did not report on this aspect.

**TABLE 4:** Characteristics of included trials of Chinese herbal formulas for rubella.

Study ID	Design type	Population	Average age (Year)	Sample size (case, T/C)	Intervention	Control	Course (d)	Outcome	Adverse reaction
Li 2012 (Li and Zeng, 2012)	RCT	Children/adult	11–28	43 (22/21)	Modified Yinqiao powder and Xijiao Dihaung decoction	Self-made prescription	3–7	Time of rash disappearance and total disease course	No
He 2008 (He et al., 2008)	RCT	Adult	T: 27.70 ± 3.44	60 (30/30)	Huanglan Granule	Western medicine antiviral therapy	20	RuV-IgM	Not reported
			C: 27.93 ± 3.33						
Zhou 1995 (Zhou, 1995)	RCT	Children	1–13	152 (122/30)	Modified Yinqiao powder	Chinese patent medicine	Not reported	Fever clearance time, time of rash disappearance, and regression time of lymphadenopathy	No

Notes: RCT, randomized controlled trial; T, treatment group: C, control group; and RuV-IgM, rubella virus-immunoglobulin M.

### General evidence of Chinese herbal formulas for measles, varicella, and rubella

**FIGURE 3:**
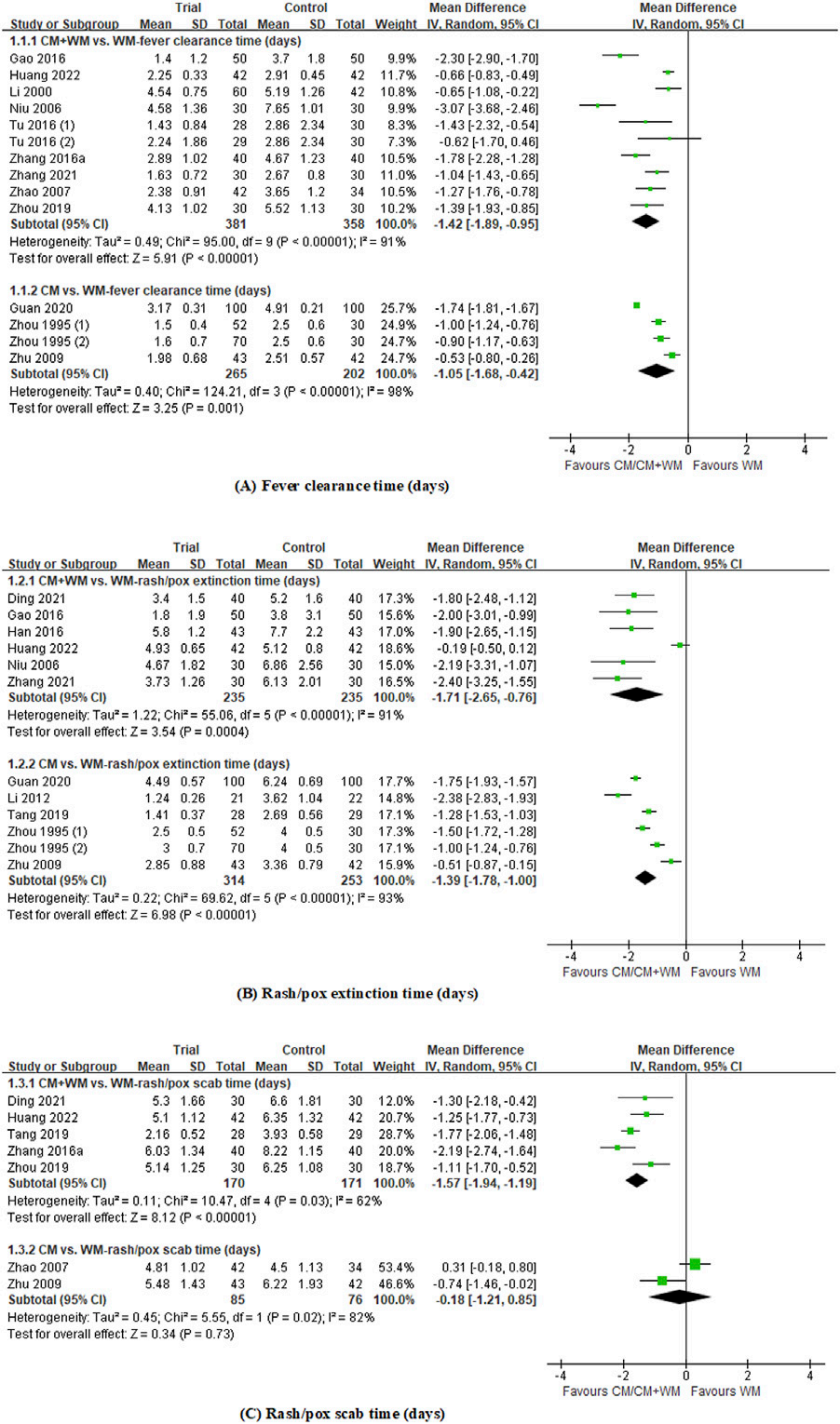
Forest plot of RCTs on CM for pox-like viral diseases on **(A)** fever clearance time; **(B)** rash/pox extinction time; **(C)** rash/pox scab time.

In summary, we identified 39 clinical trials on the treatment of measles, varicella, and rubella with CM. The data from 20 studies on fever clearance time, rash/pox extinction time, and rash/pox scab time were pooled in the meta-analysis (Figure 3). The results showed that compared with Western medicine, CM combined with Western medicine showed significant improvement in fever clearance time (MD, −1.42 days; 95% CI, −1.89 to −0.95; *p* < 0.00001; I^2^ = 91%; 10 RCTs), rash/pox extinction time (MD, −1.71 days; 95% CI, −2.65 to −0.76; *p* = 0.0004; I^2^ = 91%; six RCTs), and rash/pox scab time (MD, −1.57 days; 95% CI, −1.94 to −1.19; *p* < 0.00001; I^2^ = 62%; five RCTs). When compared with Western medicine, CM alone could fasten fever clearance (MD, −1.05 days; 95% CI, −1.68 to −0.42; *p* < 0.00001; I^2^ = 98%; 4 RCTs) and reduce the time of rash/pox extinction (MD, −1.39 days; 95% CI, −1.78 to −1.00; *p* < 0.00001; I^2^ = 93%; 6 RCTs). However, no significant difference was found in rash/pox scab time when comparing CM alone with Western medicine (MD, −0.18 days; 95% CI, −1.21 to −0.85; *p* = 0.02; I^2^ = 82%; two RCTs). In summary, whenever CM was used alone or combined with Western medicine, the symptoms of pox-like viral disease probably cleared faster when compared with Western medicine alone, lessening the time of suffering for the patients.

Of the 39 clinical trials on the treatment of measles, varicella, and rubella with Chinese medicine herbal interventions (11/39), modified Yinqiao powder (8/39), modified Xijiao Dihaung decoction (5/39), modified Qingjie Toubiao decoction, and Shengma Gegen decoction (4/39) were frequently studied and applied for pox-like viral diseases. We pooled the data from the included RCTs (Table 5) and found that when compared with Western medicine, the four formulas used alone or combined with Western medicine all showed significant differences in the reduction of fever clearance time, rash/pox extinction time, and rash/pox scab time. In summary, modified Yinqiao powder, modified Xijiao Dihaung decoction, modified Qingjie Toubiao decoction, and modified Shengma Gegen decoction could reduce the time of rash/pox extinction and scab and fever clearance.

**TABLE 5:** Meta-analysis of RCTs on four Chinese herbal formulas for contagious pox-like viral diseases.

Outcome	Comparison	No. of RCTs	No. of participant	Mean difference (95% CI)	*p*-value	I^2^
Fever clearance time (days)						
Modified Yinqiao powder (Zhou, 1995; Yang, 2003)	CM vs. WM	3	296	−0.84 [−1.07, −0.60]	*p* < 0.00001	61%
Modified Xijiao Dihaung decoction (Li, 2000)	CM + WM vs. WM	1	102	−0.65 [−1.08, −0.22]	*p* = 0.003	NA
Modified Qingjie Toubiao decoction (Niu et al., 2006)	CM + WM vs. WM	1	60	−3.07 [−3.68, −2.46]	*p* < 0.00001	NA
Modified Shengma Gegen decoction (Zhang, 2016)	CM + WM vs. WM	1	80	−1.78 [−2.28, −1.28]	*p* < 0.00001	NA
Rash/pox extinction time (days)						
Modified Yinqiao powder (Zhou, 1995; Yang, 2003)	CM vs. WM	3	296	−1.04 [−1.55, −0.53]	*p* < 0.0001	92%
Modified Xijiao Dihaung decoction (Li, 2000; Li and Zeng, 2012)	CM vs. WM	1	43	−2.38 [−2.83, −1.93]	*p* < 0.00001	NA
	CM + WM vs. WM	1	102	−0.65 [−1.08, −0.22]	*p* = 0.003	NA
Modified Qingjie Toubiao decoction (Niu et al., 2006)	CM + WM vs. WM	1	60	−2.19 [−3.31, −1.07]	*p* = 0.0001	NA
Modified Shengma Gegen decoction (Han, 2016)	CM + WM vs. WM	1	86	−1.90 [−2.65, −1.15]	*p* < 0.00001	NA
Rash/pox scab time (days)						
Modified Yinqiao powder (Yang, 2003)	CM vs. WM	1	114	−0.90 [−1.21, −0.59]	*p* < 0.00001	NA
Modified Shengma Gegen decoction (Zhang, 2016)	CM + WM vs. WM	1	80	−2.19 [−2.74, −1.64]	*p* < 0.00001	NA

Notes: RCT, randomized controlled trial; CM, Chinese medicine; WM, western medicine; and NA, not applicable.

## Discussion

The emergence of a new outbreak caused by the monkeypox virus raised concerns among public health authorities, and there were no licensed treatments for patients with monkeypox virus infection. It is urgent to investigate effective intervention strategies from CM for its prevention and treatment. Our review integrated multiple lines of evidence from historical classics and clinical studies, which suggested that modified Yinqiao powder, modified Xijiao Dihaung decoction, modified Qingjie Toubiao decoction, modified Shengma Gegen decoction, and Leiji powder were effective in preventing and treating smallpox, measles, varicella, and rubella. The evidence synthesis from other contagious pox-like viral diseases was highly significant for exploring the intervention program for monkeypox.

The multi-country outbreak of monkeypox posed unique challenges. At the time of writing, there are no specific treatments for monkeypox patients. Two oral drugs (tecovirimat and brincidofovir) have been approved for the treatment of smallpox and have demonstrated efficacy against monkeypox based on animal models, while the clinical data regarding the efficacy of these agents in humans against monkeypox were lacking (Parker et al., 2012; Adler et al., 2022). Recorded literature showed that the use of CM to prevent and treat epidemics of contagious pox-like viral diseases can be traced back to ancient CM practice over thousands of years, and its successful effects were preliminarily substantiated by modern human clinical research when applied to smallpox, measles, varicella, and rubella epidemics, which suggests that historical CM experience is a worthwhile approach.

We are worried about a new threat posed by the outbreak of monkeypox, but we still knew little about monkeypox, especially in terms of *in vivo* viral kinetics and infectivity (Learned et al., 2003; Mbala et al., 2017). The treatment and prevention of other contagious pox-like viral diseases by Chinese herbal formulas were of great significance to explore the intervention program for monkeypox. Our results suggested that compared with Western medicine, Chinese herbal formulas combined with Western medicine and Chinese herbal formulas alone both showed significant improvements in fever clearance time, rash/pox extinction time, and rash/pox scab time, which indicates that the symptoms of contagious pox-like viral diseases may improve faster than those of Western medicine alone, especially in reducing time rash/pox extinction and fever clearance. The most frequently used Chinese herbal formulas included modified Yinqiao powder, modified Xijiao Dihaung decoction, modified Qingjie Toubiao decoction, modified Shengma Gegen decoction, and Leiji powder. The use of Chinese herbal formulas to prevent and treat other contagious pox-like viral diseases may be effective in treating monkeypox infections.

Yinqiao powder came from the *Treatise on differentiation and treatment of epidemic febrile disease* in the Qing Dynasty. Yinqiao powder was a classic prescription for treating exothermic febrile diseases with clinical symptoms including fever and sore throat. It has the effect of releasing the exterior with pungent cool, clearing heat, and removing toxins, mainly used in the early stage of epidemic febrile diseases. The core components of Yinqiao powder, *Lonicera japonica* Thunb. and *Forsythia suspensa* (Thunb.) Vahl were the most powerful traditional medicines for heat-clearing and detoxification. The other components including *Platycodon grandiflorus* (Jacq.) A. DC., *Mentha canadensis* L., *Glycyrrhiza glabra* L., and *Arctium lappa* L. have effects for clearing the throat. *Sesamum indicum* L. and *Glycine* max L.) Merr. have the effects of inducing sweating to release the exterior. These medicines were used together to give Yinqiao powder the ability to release the exterior with pungent cool, clear heat, and remove toxins (Wang et al., 2011). At the initial stage of febrile disease infection, Yinqiao powder has a strong effect of blocking the progress of the disease. In China, Yinqiao powder was widely used to treat viral pneumonia, COVID-19, measles, varicella, and rubella (Lyu et al., 2020; Xu and Jia, 2020; Guo et al., 2021a; Lin et al., 2021). An experimental study found that the effect of Yinqiao powder in preventing and treating upper respiratory tract infection could be explained by its antibacterial and antiviral properties and improvement of the function of the upper respiratory mucosal immune system (Liu et al., 2015). A multicenter, large-scale, randomized trial found that Yinqiao powder plus another heat-clearing formula could reduce time to fever resolution in patients with the H1N1 influenza virus infection (Wang et al., 2011). Pharmacological studies have shown that Yinqiao powder has an antitussive and expectorant effect, improving lung function, alleviating acute lung injury, alleviating pulmonary fibrosis, enhancing the antiviral immune response, and alleviating the adverse effects of modern drugs (Rothan and Byrareddy, 2020). The monkeypox identified during the 2022 outbreak begins with non-specific symptoms and signs that include fever, headache, muscle aches, back pain, low energy, and swollen lymph nodes; it can also begin with a fever before rashes appear which may last for 2 to 3 weeks (Petersen et al., 2019). Our results found that Yinqiao powder could improve the time of rash/pox extinction, rash/pox scab, and fever clearance.

Shengma Gegen decoction was a classic prescription to treat pox-like viral diseases coming from the *Formulary of the Bureau of Taiping People’s Welfare Pharmacy* in the Song Dynasty, which was composed of *A. cimicifuga* L., *Pueraria montana* var. *lobata* (Willd.) Maesen and S.M.Almeida ex Sanjappa and Predeep, *Paeonia lactiflora* Pall., and *G. glabra* L. This prescription has an outstanding effect of releasing flesh with pungent cool, removing toxins, and promoting eruption, and it was mainly used for measles, varicella, rubella and other eruptive diseases, viral pneumonia, herpes, and hepatitis C (Zhu et al., 1989; Niu, 1996; Li et al., 2015). Pharmacological research proved that *A. cimicifuga* L. and *P. montana* var. *lobata* (Willd.) Maesen and S.M.Almeida ex Sanjappa and Predeep exhibited anti-osteoporosis, antiviral, antitumor, antioxidant, and antiangiogenic activities (Guo et al., 2017; Hu et al., 2021). The main effect of this prescription was releasing exterior and promoting eruption. In clinical application, it can be used together with botanical drug *M. canadensis L.*, *L. japonica* Thunb., and *A. lappa* L. to enhance the power of promoting eruption and clearing heat. In the incubation period of monkeypox disease or when clinical symptoms such as fever and rash appear, Yinqiao powder may be effective in improving clinical symptoms. Therefore, Shengma Gegen decoction could be combined with Yinqiao powder to treat monkeypox during the incubation period or when the patient has fever symptoms and the rash does not appear completely. Meanwhile, Shengma Gegen decoction has been officially recommended in the guidelines for the diagnosis and treatment of monkeypox (2022 version) (Interpretation of guidelines for diagnosis, 2022) by the National Health Commission of the People’s Republic of China.

Modified Qingjie Toubiao decoction, a famous empirical prescription, comes from the *Dictionary of Traditional Chinese Medicine*. When measles patients have symptoms such as intensive measles, persistent high fever, and sore throat, Qingjie Toubiao decoction can be used to clear heat and detoxify and promote the penetration of rash (ang, 1995). Qingjie Toubiao decoction has the effect of expelling pathogens through the exterior with pungent cool and ventilating lung, which has been proven to be effective in the clinical practice of CM. *Tamarix chinensis* Lour. has effects of exterior releasing and promoting eruption, expelling wind, and eliminating dampness. *Pterocarpus lucens* Lepr. ex Guill. and Perr. and *A. lappa* L. could promote eruption and clear heat. *Actaea cimicifuga* L. and *P. montana* var. *lobata* (Willd.) Maesen and S.M.Almeida ex Sanjappa and Predeep could release flesh and promote eruption. Compared with Shengma Gegen decoction, Qingjie Toubiao decoction was more effective in removing toxins, relieving symptoms, cooling blood, and relieving itching. It might be used in the early, middle, and even the late stages of monkeypox infections and was of great help in relieving patients’ clinical symptoms. Pharmacological research showed that *F. suspensa* (Thunb.) Vahl, *Morus alba* L., and *L. japonica* Thunb. have the effects of clearing heat and removing toxins. *Pueraria montana* var. *lobata* (Willd.) Maesen and S.M.Almeida ex Sanjappa and Predeep, *P. lucens* Lepr. ex Guill. and Perr., *A. lappa* L., and *T. chinensis* Lour. could help in exterior releasing, ventilating lung, promoting eruption, and relieving itching. *Alternanthera sessilis* L.) R. Br. ex DC*.* could be used at any stage of rash outbreak and could clear heat and cool blood, remove toxins, and promote eruption. Qingjie Toubiao decoction could effectively control body temperature, promote the appearance of skin rash, reduce serious complications, shorten the course of the disease, and improve the curative effect when used at the eruptive stage of contagious pox-like viral diseases.

Modified Xijiao Dihaung decoction was a classical prescription of CM derived from *Important Prescriptions Worth a Thousand Gold for Emergency* by Sun Simiao in the Tang Dynasty. Xijiao Dihaung decoction contains *Cornu Bubali*, *Rehmannia glutinosa* (Gaertn.) DC., *P. lactiflora* Pall., and *Paeonia × suffruticosa* Andrews. Xijiao Dihaung decoction has the effect of nourishing yin, reducing fever, dispelling phlegm, and cooling the blood and was recommended to patients who suffered from pneumonia, alimentary tract hemorrhage, radiation enteritis, and sepsis. Animal experiments showed that Yinqiao powder combined with Xijiao Dihaung decoction has a therapeutic effect on influenza A virus infection, and the underlying mechanisms might be related to regulating antiviral immunity and ameliorating excessive inflammatory responses (Li et al., 2020). A pharmacological study also suggested that Xijiao Dihaung decoction combined with Yinqiao powder inhibits the inflammatory response mediated by the MAPK signaling pathway to treat influenza viral pneumonia (Guo et al., 2021a). Our review showed that Xijiao Dihaung decoction was mainly used for measles, varicella, and rubella patients with severe clinical symptoms, such as persistent fever, skin rashes that do not scab, pneumonia, and other complications. For monkeypox patients, Xijiao Dihaung decoction may promote the crusting of patients’ skin rashes and shorten the fever time.

People who lived with or had close contact (including sexual contact) with someone who has monkeypox are most at risk. Currently, there are three smallpox vaccines in the US Strategic National Stockpile: JYNNEOS and ACAM2000 are licensed for smallpox, which may be useful for monkeypox (Rizk et al., 2022). While the safety information on vaccines (such as ACAM200) among children or pregnant women is limited, the vaccine efficacy/effectiveness against monkeypox in the current epidemic remains uncertain. Our results showed that Leiji powder had a significant effect on the prevention of pox-like viral diseases; Leiji powder comes from the *Xiaoer Weisheng Zongwei Lunfang* in the Southern Song Dynasty. Historically, there were a lot of reports on the use of Leiji powder to prevent and treat measles, scarlatina, cholera, diarrhea, jaundice, dysentery, syncope, and other diseases in China (Li and Li, 1959; Zhang, 1960; Zheng, 1960; Chen, 1964; Yu, 1996). We searched nine literature reports on the use of Leiji powder (nasal drip) to prevent measles. There was a report on the use of Leiji powder to prevent measles in 4,468 measles-susceptible children, with an incidence rate of 14.5% (Zheng, 1960). Another non-RCT including 2,031 subjects, reported that the incidence rate was 17.2% (Jinzhou city hospital, 1959). The nasal drip of Leiji powder was effective in preventing measles, which could significantly reduce the severity of the disease, shorten the course of the disease, reduce complications, and have no adverse reactions (Zhang, 1960; Lu, 1957; Qian et al., 1959; Measles prevention and control group of Shengli road office of Xinxiang City, 1959). Although one RCT study (Chen, 1964) (n = 103) showed that Leijisan had no effect on measles prevention, it might be related to the way of drug production, method used, and treatment course (Qian et al., 1959; Measles prevention and control group of Shengli road office of Xinxiang City, 1959). The main metabolite *Gleditsia sinensis* Lam. of Leiji powder has the function of resolving phlegm for resuscitation, reducing swelling, and alleviating pain (Dong et al., 2022). *Angelica dahurica* (Hoffm.) Benth. and Hook. f. ex Franch. and Sav. and *Atractylodes lancea* (Thunb.) DC. have antiviral and antibacterial effects and were effective in preventing measles and influenza (Qian et al., 1959; Huang et al., 2022). Another metabolite, *Liquidambar orientalis* Mill., could resolve dampness and remove toxins. Modern pharmacological studies have proven that *L. orientalis* Mill. possesses potent anti-inflammatory effects, neuroprotective effects, anti-cancer effects, and antioxidant effects (Guo et al., 2021b; Liu et al., 2021). The components of this prescription can promote the disappearance of herpes in patients with severe herpes eruptions. More prospective population studies are needed to further investigate the effect of Leiji powder in preventing monkeypox.

## Limitations

Our study had several limitations. Human monkeypox was first reported in central Africa in 1970 (Marennikova et al., 1972) and has never broken out in Chinese history. There were no data on using CM for monkeypox in historical records and human research. However, there are many studies on the use of CM to prevent and treat contagious pox-like viral diseases in China. The suggestions from other pox-like viral diseases may be beneficial for exploring the appropriate solutions for monkeypox. Second, we searched records of using CM for contagious pox-like viral diseases including monkeypox, smallpox, measles, varicella, and rubella in the review. It might not be completely representative of respiratory viral diseases. Finally, as there was no direct clinical evidence for the prevention and treatment of the new breakout monkeypox, currently reported studies were from the previous literature on other contagious pox-like viral diseases which can only be considered indirect evidence to refer to the current outbreak. There is an urgent need for prospective studies with rigorous design and a large sample to evaluate the effect of Chinese herbal formulas on monkeypox infections.

## Conclusion

In conclusion, based on historical records and clinical studies of contagious pox-like viral diseases, the Chinese herbal formulas could be considered an alternative approach for the prevention and treatment of monkeypox. According to the principle of syndrome differentiation and treatment of CM, Yinqiao powder, Shengma Gegen decoction, Qingjie Toubiao decoction, and Xijiao Dihaung decoction had the potential to be useful for monkeypox treatment. Leiji powder may be effective in preventing monkeypox infection. The main limitation of this study is that there was no direct clinical evidence of the treatment of monkeypox with CM. We look forward to real-world studies to verify our hypothesis. Furthermore, prospective well-designed clinical studies are needed to investigate the effectiveness of Chinese herbal formulas for monkeypox.

## Data Availability

The original contributions presented in the study are included in the article/Supplementary Material, further inquiries can be directed to the corresponding author.
