# Supplementary material for: Evidence synthesis of Chinese medicine for monkeypox: Suggestions from other contagious pox-like viral diseases
Source: Front Pharmacol. 2023 Mar 13;14:1121580. doi: 10.3389/fphar.2023.1121580 (PMC10040637; doi:10.3389/fphar.2023.1121580)
Supplement: Supplementary file 1 [file DataSheet1.PDF]

Supplement

eTable 1. Characteristics of included trials of herbal formulae for measles

eTable 2. Characteristics of included trials of herbal formulae for varicella

eTable 3. Characteristics of included trials of herbal formulae for rubella

eTable 1. Characteristics of included trials of herbal formulae for measles

Study ID	Chinese medicine diagnostic criteria	Western medicine diagnostic criteria	Therapeutic principles and methods	Intervention	Herbal formulae
Xu 2020	Not report	Not report	Clearing heat, removing toxin	Modified Yinqiao powder	*Strobilanthes cusia* (Nees) Kuntze 10g, *Forsythia suspensa* (Thunb.) Vahl 10g, *Phragmites australis* (Cav.) Trin. ex Steud. 10g, *Lonicera japonica* Thunb. 10g, *Bupleurum chinense* DC. 6g, *Morus alba* L. 6g, *Ricinus communis* L. 6g, *Scrophularia ningpoensis* Hemsl. 6g, *Gardenia jasminoides* J.Ellis 5g, *Scutellaria baicalensis* Georgi 3g, *Prunus armeniaca* L. 3g, *Mentha canadensis* L. 3g.
Zhou 2019	Not report	Zhu Futang Practice of Pediatrics[M]. 2015	Promoting eruption and expelling pathogen from exterior, removing toxin with cooling therapy	Self-made prescription	*Rehmannia glutinosa* (Gaertn.) DC. 15g, *Arnebia euchroma* (Royle ex Benth.) I.M.Johnst. 10g, *Taraxacum mongolicum* Hand.-Mazz. 15g, *Pterocarpus lucens* Lepr. ex Guill. & Perr. 10g, *Nepeta cataria* L. 10g, *Mentha canadensis* L. 10g.
Tu 2016	Not report	Not report	Expelling pathogen through exterior, removing toxin, nourishing yin	Modified Qingjie Toubiao decoction	T1: *Strobilanthes cusia* (Nees) Kuntze; T2: *Lonicera japonica* Thunb. 5-30g, *Scutellaria baicalensis* Georgi 5-20g, *Gardenia jasminoides* J.Ellis 5-30g, *Isatis tinctoria* subsp. Tinctorial 5-20g, *Ricinus communis* L. 5-15g, *Lophatherum gracile* Brongn. 5-10g, *Arnebia euchroma* (Royle ex Benth.) I.M.Johnst. 10-30g, *Phragmites australis* (Cav.) Trin. ex Steud. 10-30g, *Arctium lappa* L. 5-15g, *Radix Platycodon grandiflorus* (Jacq.) A.DC 5-15g, *Pterocarpus lucens* Lepr. ex Guill. & Perr. 5-10g, *Glycyrrhiza glabra* L. 10-20g.
Li 2015	Not report	Not report	Removing toxin with cooling therapy, nourishing yin	Self-made prescription	*Artemisia annua* L. 10g, *Nepeta cataria* L. 10g, *Mentha canadensis* L. 10g, *Curcuma longa* L 10g.
Liu 2013	Not report	Infectious Diseases[M]. 2004	Inducing sweating and promoting eruption, clearing heat and relieving itching, cooling blood and removing toxin	Self-made prescription	*Coriandrum sativum* L. 100 g, *Artemisia annua* L. 20g, *Spirodela polyrhiza* (L.) Schleid. 20g, *Perilla frutescens* (L.) Britton 20g, *Mentha canadensis* L. 20g.
Zhang 2009	Not report	Diagnostic criteria and principles of management of measles (GB15983-1995) [S]. 2004	Dispel evil heat	Modified Huanglian Jiedu decoction, Modified Xijiao Dihaung decoction	*Gardenia jasminoides* J.Ellis 10g, *Coptis chinensis* Franch. 5g, *Scutellaria baicalensis* Georgi 10g, *Rehmannia glutinosa* (Gaertn.) DC. 15g, *Paeonia lactiflora* Pall. 15g, *Forsythia suspensa* (Thunb.) Vahl 15g.
Niu 2006	Traditional Chinese Medicine Pediatrics[M]. 1989	Practice of Infectious Diseases[M]. 1998	Ventilating lung, removing toxin, promoting eruption	Modified Qingjie Toubiao decoction	*Cornu Saigae Tataricae, Pterocarpus lucens* Lepr. ex Guill. & Perr., *Pueraria montana var. lobata* (Willd.) Maesen & S.M.Almeida ex Sanjappa & Predeep, *Actaea cimicifuga* L., *Arnebia euchroma* (Royle ex Benth.) I.M.Johnst., *Forsythia suspensa* (Thunb.) Vahl, *Paeonia lactiflora* Pall., *Ricinus communis* L., *Chrysanthemum × morifolium* (Ramat.) Hemsl., *Saposhnikovia divaricata* (Turcz. ex Ledeb.) Schischk. Modified based on different symptoms or signs.
Zhang 2004	Not report	Not report	Ventilating lung and discharging heat with pungent-cool, clearing lung and relieving asthma	Modified Maxing Shigan decoction	*Rehmannia glutinosa* (Gaertn.) DC. 4-9g, *Prunus armeniaca* L. [Rosaceae] 5-12g, *Glycyrrhiza glabra* L. 4-9g, *Gypsum* 12-25g. Modified based on different symptoms or signs.
Li 2000	Diagnosis and differential diagnosis of Chinese Medicine[M]. 1999	Practice of Infectious Diseases[M]. 1998	Clearing heat, removing toxin, promoting eruption	Modified Xijiao Dihaung decoction	*Cornu Bubali* 15-30g, *Forsythia suspensa* (Thunb.) Vahl 6g, *Radix Platycodon grandiflorus* (Jacq.) A.DC 4g, *Rehmannia glutinosa* (Gaertn.) DC. 12g, *Paeonia lactiflora* Pall. 6g, *Scutellaria baicalensis* Georgi 6g, *Arnebia euchroma* (Royle ex Benth.) I.M.Johnst. 6g, *Taraxacum mongolicum* Hand.-Mazz. 9g. Modified based on different symptoms or signs.

Wang 1995	Not report	Not report	Clearing heat and removing toxin, expelling pathogen through exterior	Modified Qingjie Toubiao decoction, Modified Maxing Shigan decoction	Modified Qingjie Toubiao Decoction: *Tamarix chinensis* Lour., *Pterocarpus lucens* Lepr. ex Guill. & Perr., Maesen & S.M.Almeida ex Sanjappa & Predeep, *Arnebia euchroma* (Royle ex Benth.) I.M.Johnst., *Ricinus communis* L., *Chrysanthemum × morifolium* (Ramat.) Hemsl., *Glycyrrhiza glabra* L., *Arctium lappa* L., *Lonicera japonica* Thunb., *Forsythia suspensa* (Thunb.) Vahl 6g, *Gypsum*, *Rehmannia glutinosa* (Gaertn.) DC., *Dendrobium nobile* Lindl.. Modified Maxing Shigan decoction: *Rehmannia glutinosa* (Gaertn.) DC., *Prunus armeniaca* L. 3, *Gypsum*, *Glycyrrhiza glabra* L., *Pterocarpus lucens* Lepr. ex Guill. & Perr., *Fritillaria thunbergii* Miq., *Kitagawia praeruptora* (Dunn) Pimeno, *Lonicera japonica* Thunb., *Radix Platycodon grandiflorus* (Jacq.) A.DC, *Bambusa textilis* McClure.
Zhao 2021	Not report	Zhu Futang Practice of Pediatrics[M]. 1995	Clearing heat and removing toxin, purging heat and relieve constipation	Modified Xuanbai Chengqi decoction	*Prunus armeniaca* L. 10g, *Trichosanthes kirilowii* Maxim. 10g, *Gypsum* 15-30g, *Rheum officinale* Baill. 5-10g. Modified based on different symptoms or signs.
Ye 1962	Not report	Not report	Expelling pathogen through exterior, clearing heat, nourishing yin	Maxing Shigan decoction, Modified Yinqiao powder, Modified Qianjin Weijing decoction	Maxing Shigan decoction: *Ricinus communis* L. 2.2g, *Chrysanthemum × morifolium* (Ramat.) Hemsl. 2.2g, *Platycodon grandiflorus* (Jacq.) A.DC. 4.7g, *Forsythia suspensa* (Thunb.) Vahl 9.4g, *Lophatherum gracile* Brongn. 9.4g, *Coriandrum sativum* L. 0.9g, *Glycyrrhiza glabra* L. 3.1g. Modified Yinqiao powder: *Lonicera japonica* Thunb. 9.4g, *Glycine max* (L.) Merr. 6.3g, *Forsythia suspensa* (Thunb.) Vahl 9.4g, *Lophatherum gracile* Brongn. 15.6g, *Platycodon grandiflorus* (Jacq.) A.DC. 6.3g, *Glycyrrhiza glabra* L.9.4g, *Rehmannia glutinosa* (Gaertn.) DC. 2.2g, *Gardenia jasminoides* J.Ellis 6.3g. Modified Qianjin Weijing decoction: *Phragmites australis subsp. Australis* 18.7g, *Lonicera japonica* Thunb. 9.4g, *Platycodon grandiflorus* (Jacq.) A.DC. 6.3g, *Isatis tinctoria subsp. Tinctorial* 9.4g, *Lophatherum gracile Brongn*. 15.6g, *Yuquan powder* 18.8g, *Ophiopogon japonicus* (Thunb.) Ker Gawl. 9.4g, *Adenophora triphylla* (Thunb.) A.DC. 9.4g.
303 Hospital 1959	Not report	Not report	Promoting eruption, removing toxin, nourishing yin	Zicao Fuping decoction, Sanhaung Shigao decoction	Zicao Fuping decoction: *Arnebia euchroma* (Royle ex Benth.) I.M.Johnst. 3.1g, *Spirodela polyrhiza* (L.) Schleid. 3.1g, *Pterocarpus lucens* Lepr. ex Guill. & Perr. 3.1g, *Carthamus tinctorius* L. 1.6g, *Pueraria montana var. lobata* (Willd.) Maesen & S.M.Almeida ex Sanjappa & Predeep 3.1g, *Bassia scoparia* (L.) A.J.Scott 3.1g. Sanhaung Shigao decoction: *Rehmannia glutinosa* (Gaertn.) DC. 3.1g, *Scutellaria baicalensis* Georgi 4.7g, *Gardenia jasminoides* J.Ellis 4.7g, *Coptis chinensis* Franch. 6.3g, *Phellodendron amurense* Rupr. 6.3g, *Gypsum* 9.4g, *Glycine max* (L.) Merr. 9.4g.
Qi 2016	Not report	Practice of Internal Medicine[M]. 2005	Clearing heat, removing toxin, promoting eruption	Modified Shengma Gegen decoction	*Actaea cimicifuga* L. 30g, *Paeonia lactiflora* Pall 30g, *Glycyrrhiza glabra* L. 30g, *Pueraria montana var. lobata* (Willd.) Maesen & S.M.Almeida ex Sanjappa & Predeep 45g, *Lonicera japonica* Thunb. 15g, *Forsythia suspensa* (Thunb.) Vahl 15g.

eTable 2. Characteristics of included trials of herbal formulae for varicella

Study ID	Chinese medicine diagnostic criteria	Western medicine diagnostic criteria	Therapeutic principles and methods	Intervention	Herbal formulae
Huang 2022	Clinical research on pediatrics of Chinese Medicine[M]. 2009	Zhu Futang Practice of Pediatrics[M]. 2002	Clearing qi aspect and cooling nutrient aspect, removing toxin and resolving dampness	Huanglian Jiedu decoction, Xijiao Dihaung decoction, Modified Six-one powder	*Coptis chinensis* Franch. 20g, *Scutellaria baicalensis* Georgi 20g, *Rehmannia glutinosa* (Gaertn.) DC. 15g, *Forsythia suspensa* (Thunb.) Vahl 15g, *Actaea cimicifuga* L. 12g, *Paeonia × suffruticosa Andrews* 12g, *Paeonia lactiflora* Pall. 12g, *Arnebia euchroma* (Royle ex Benth.) I.M.Johnst. 10g, *Gypsum*10g, *Gardenia jasminoides* J.Ellis 10g, *Talcum* 10g, *Strobilanthes cusia* (Nees) Kuntze 10g, *Mangifera indica* L. 10g, *Plantago asiatica* L. 6g, *Glycyrrhiza glabra* L. 6g.
Zhang 2021	Guideline for clinical diagnosis and treatment of pediatrics of Chinese medicine· varicella (Amendment)	Pediatrics[M]. 2017	Dispersing wind and clearing heat, removing toxin and draining dampness	Self-made prescription	*Angelica dahurica* (Hoffm.) Benth. & Hook.f. ex Franch. & Sav. 30g, *Atractylodes lancea* (Thunb.) DC. 30g, *Bassia scoparia* (L.) A.J.Scott 30g, *Lonicera japonica* Thunb. 40g, *Spirodela polyrhiza* (L.) Schleid. 20g, *Pterocarpus lucens* Lepr. ex Guill. & Perr. 20g, *Sophora flavescens* Aiton 30g.
Din 2021	Traditional Chinese Medicine Pediatrics[M]. 2021	Not report	Clearing heat, removing toxin, draining dampness	Self-made prescription	*Sophora flavescens* Aiton 20g, *Phellodendron amurense* Rupr. 20g, *Rheum officinale* Baill. 20g, *Dictamnus dasycarpus* Turcz. 10g, *Portulaca oleracea* L. 10g.
Guan 2020	Not report	Not report	Clearing heat and removing toxin, draining dampness and eliminating turbid	Yinqiao powder, Three-nut decoction	*Lonicera japonica* Thunb. 15g, *Forsythia suspensa* (Thunb.) Vahl 9g, *Pterocarpus lucens* Lepr. ex Guill. & Perr. 9g, *Lophatherum gracile* Brongn. 15g, *Oroxylum indicum* (L.) Kurz 10g, *Glycine max* (L.) Merr. 10g, *Strobilanthes cusia* (Nees) Kuntze 15g, *Platycodon grandiflorus* (Jacq.) A.DC. 6g, *Phragmites australis* (Cav.) Trin. ex Steud. 10g, *Coix lacryma-jobi* L. 15g, *Talcum* 9g, *Myristica fragrans* Houtt. 10g, *Plantago asiatica* L. 6g, *Magnolia officinalis* Rehder & E.H.Wilson 6g, *Taraxacum mongolicum* Hand.-Mazz. 10g, *Glycyrrhiza glabra* L. 3g. Modified based on different symptoms or signs.
Tang 2019	The Standard for TC M Diseases and Syn dromes therapeutic r esults[S]. 1995	Not report	Removing pestilential toxin and removing toxin, cooling the blood and eliminating the pox	Lianhua Qingwen Capsules	*Forsythia suspensa* (Thunb.) Vahl 255g, *Lonicera japonica* Thunb. 255g, *Rehmannia glutinosa* (Gaertn.) DC. 85g, *Prunus armeniaca* L. 85g, *Cypsum* 255g, *Strobilanthes cusia* (Nees) Kuntze 255g, *Cyrtomium fortunei* J.Sm. 255g, *Houttuynia cordata* Thunb. 255g, *Pogostemon cablin* (Blanco) Benth. 85g, *Rheum officinale* Baill. 51g, *Rhodiola crenulata* (Hook.f. & Thomson) H.Ohba 85g, *Mentha canadensis* L. 7.5g, *Glycyrrhiza glabra* L.85g.
Gao 2016	Not report	Not report	Clearing heat and removing toxin	Modified Yinqiao powder, Modified Six-one powder	*Ricinus communis* L., *Chrysanthemum × morifolium* (Ramat.) Hemsl., *Lonicera japonica* Thunb., *Forsythia suspensa* (Thunb.) Vahl, *Pterocarpus lucens* Lepr. ex Guill. & Perr., *Fritillaria cirrhosa* D.Don, *Prunus armeniaca* L., *Arctium lappa* L., *Bombyx Batryticatus*, *Nepeta cataria* L., *Glycine max* (L.) Merr., *Mentha canadensis* L., *Platycodon grandiflorus* (Jacq.) A.DC., *Lophatherum gracile* Brongn., Trin. ex Steud., *Talcum*, *Glycyrrhiza glabra* L.
Zhang 2016a	Not report	Diagnostic and therapeutic criteria for varicella[S]	Clearing heat and cooling blood, removing toxin and removing dampness	Shengma Gegen decoction, White Tiger decoction, Xijiao Dihaung decoction	*Glycyrrhiza glabra* L. 6g, *Arnebia euchroma* (Royle ex Benth.) I.M.Johnst. 6g, *Paeonia × suffruticosa Andrews* 6g, *Cornu Bubali* 6g, *Spirodela polyrhiza* (L.) Schleid.10g, *Phragmites australis* (Cav.) Trin. ex Steud.10g, *Actaea cimicifuga* L. 10g, *Arctium lappa* L. 10g, *Anemarrhena asphodeloides* Bunge 10g, *Crataegus pinnatifida* Bunge 12g, *Cypsum* 15g.
Han 2016	Not report	Pediatrics[M]. 2004	Clearing heat and cooling blood, removing dampness and removing toxin	Shengma Gegen decoction, Baihu decoction, Xijiao Dihaung decoction	*Actaea cimicifuga* L.10g, *Arnebia euchroma* (Royle ex Benth.) I.M.Johnst. 6g, *Cornu Bubali* 6g, *Paeonia × suffruticosa Andrews* 6g, Schleid. 10g, *Arctium lappa* L. 10g, *Pueraria montana var. lobata* (Willd.) Maesen & S.M.Almeida ex Sanjappa & Predeep 10g, *Anemarrhena asphodeloides* Bunge 10g, *Crataegus pinnatifida* Bunge 12g, *Cypsum* 15g, *Glycyrrhiza glabra* L. 6g.

Wang 2015	Handbook of Chinese medicine practitioners[M]. 2000	Not report	Clearing heat and removing toxin, dispersing wind and relieving itching, promoting urination and removing dampness	Modified Wuli Huichun pills	*Forsythia suspensa* (Thunb.) Vahl., *Scutellaria baicalensis* Georgi, *Isatis tinctoria* subsp. Tinctorial, *Lophatherum gracile* Brongn., *Pueraria montana var. lobata* (Willd.) Maesen & S.M.Almeida ex Sanjappa & Predeep, *Bupleurum chinense* DC., *Arctium lappa* L., *Pterocarpus lucens* Lepr. ex Guill. & Perr., *Talcum*, *Plantago asiatica* L., *Glycine max* (L.) Merr., *Bassia scoparia* (L.) A.J.Scott, *Nepeta cataria* L., *Platycodon grandiflorus* (Jacq.) A.DC., Trin. ex Steud., *Glycyrrhiza glabra* L.. Modified based on different symptoms or signs.
Quan 2011	Not report	Pediatrics[M]. 2004	Clearing heat and removing toxin	Kushen decoction	*Sophora flavescens* Aiton 15g, *Bassia scoparia* (L.) A.J.Scott 15g, *Rheum officinale* Baill. 15g, *Lonicera japonica* Thunb. 15g, *Houttuynia cordata* Thunb. 15g, *Cnidium monnieri* (L.) Cusson 10g, *Dictamnus dasycarpus* Turcz. 10g, *Pterocarpus lucens* Lepr. ex Guill. & Perr. 10g, *Phellodendron amurense* Rupr. 10g.
Chen 2010	Not report	Not report	Clearing heat and removing toxin, draining dampness, ventilating lung and resolving phlegm	Modified Yinqiao powder	*Lonicera japonica* Thunb. 5g, *Forsythia suspensa* (Thunb.) Vahl 5g, *Lophatherum gracile* Brongn. 5g, *Mentha canadensis* L. 5g, *Arctium lappa* L. 5g, *Platycodon grandiflorus* (Jacq.) A.DC. 5g, *Pterocarpus lucens* Lepr. ex Guill. & Perr. 5g, *Cypsum* 10g, *Plantago asiatica* L. 5g.
Zeng 2009	Not report	Not report	Clearing heat and cooling blood, removing toxin and dampness	Modified Yinqiao powder, Modified Qingwen Baidu decoction	Modified Yinqiao powder: *Lonicera japonica* Thunb. 12g, *Forsythia suspensa* (Thunb.) Vahl 12g, *Platycodon grandiflorus* (Jacq.) A.DC. 9g, *Mentha canadensis* L. 3g, *Bombyx Batryticatus*, *Nepeta cataria* L., 6g, *Taraxacum mongolicum* Hand.-Mazz. 12g, *Lophatherum gracile* Brongn 9g, *Morus alba* L. 9g, *Glycyrrhiza glabra* L. 5g, *Phragmites australis* (Cav.) Trin. ex Steud. 15g, *Ricinus communis* L. 12g, *Chrysanthemum × morifolium* (Ramat.) Hemsl. 9g, *Akebia trifoliata* (Thunb.) Koidz. 3g, *Scutellaria baicalensis* Georgi 9g, *Elephantopus scaber* L. 6g.<br>Modified Qingwen Baidu decoction: *Cypsum* 180-240*g (large formula), Cypsum* 60-120g (mild formula), *Cypsum* 24-36g (small formula), *Rehmannia glutinosa (Gaertn.)* DC. 18-30g (large formula), *Rehmannia glutinosa (Gaertn.)* DC. 9-15g (mild formula), *Rehmannia glutinosa (Gaertn.)* DC. 6-13.5g (small formula), *Rhinoceros unicornis* L. 18-24g (large formula), *Rhinoceros unicornis* L. 9-12g (mild formula), *Rhinoceros unicornis* L. 6-12g (small formula), *Coptis chinensis* Franch. 12-18g (large formula), *Coptis chinensis* Franch. 6-12g (mild formula), *Coptis chinensis* Franch. 3-4.5g (small formula), *Gardenia jasminoides* J.Ellis, *Platycodon grandiflorus* (Jacq.) A.DC., *Scutellaria baicalensis* Georgi, *Anemarrhena asphodeloides* Bunge, *Forsythia suspensa* (Thunb.) Vahl, *Paeonia × suffruticosa Andrew*, *Paeonia lactiflora* Pall., *Glycyrrhiza glabra* L., Scrophularia ningpoensis Hemsl., *Lophatherum gracile* Brongn..
Zhu 2009	Not report	Zhu Futang Practice of Pediatrics[M]. 2002	Dispersing wind and clearing heat, removing toxin and dampness	Modified Yinqiao powder, Modified Three-nut decoction	*Lonicera japonica* Thunb. 10g, *Forsythia suspensa* (Thunb.) Vahl. 10g, *Arctium lappa* L. 10g, *Artemisia capillaris* Thunb. 10g, *Paeonia lactiflora* Pall. 10g, *Celosia argentea* L. 10g, *Coix lacryma-jobi* L. 10g, *Pogostemon cablin* (Blanco) Benth. 6g, *Scutellaria baicalensis* Georgi 6g, *Wurfbainia vera (Blackw.) Škorničk. & A.D.Poulsen* 3g. Modified based on different symptoms or signs.
Zhao 2009	Not report	Zhu Futang Practice of Pediatrics[M]. 2005	Releasing exterior with pungent-cool, clearing heat and removing toxin	Modified Qingjie Toubiao decoction	*Lonicera japonica* Thunb. 30g, *Forsythia suspensa* (Thunb.) Vahl 15g, *Coptis chinensis* Franch. 10g, *Ricinus communis* L. 15g, *Strobilanthes cusia* (Nees) Kuntze 30g, *Corydalis bungeana* Turcz. 15g, *Pterocarpus lucens* Lepr. ex Guill. & Perr. 15g, *Paeonia lactiflora* Pall. 15g, *Akebia trifoliata* (Thunb.) Koidz. 10g, *Chrysanthemum × morifolium* (Ramat.) Hemsl. 15g, *Glycyrrhiza glabra* L. 6g.
Zhao 2007	Not report	Zhu Futang Practice of Pediatrics[M]. 2005	Clearing heat and cooling blood, removing toxin and dampness	Modified Qingjie Toubiao decoction, Modified Wuwei Xiaodu decoction	*Ricinus communis* L., *Lonicera japonica* Thunb., *Lonicera japonica* Thunb., *Coptis chinensis* Franch., *Strobilanthes cusia* (Nees) Kuntze, *Corydalis bungeana* Turcz., *Pterocarpus lucens* Lepr. ex Guill. & Perr., *Paeonia lactiflora* Pall., *Akebia trifoliata* (Thunb.) Koidz., *Chrysanthemum × morifolium* (Ramat.) Hemsl..
Chen 2006	Not report	China Clinical Dermatology[M]. 1992	Clearing heat and cooling blood, removing toxin and draining dampness	Modified Youlong decoction	*Lonicera japonica* Thunb. 18g, *Cypsum* 18g, *Pterocarpus lucens* Lepr. ex Guill. & Perr. 10g, *Arctium lappa* L. 10g, *Strobilanthes cusia* (Nees) Kuntze 30g, *Paris polyphylla* Sm. 10g, *Arnebia euchroma* (Royle ex Benth.) I.M.Johnst. 10g, *Scutellaria baicalensis* Georgi 10g, *Dioscorea collettii var.*

					hypoglauca (Palib.) S.J.Pei & C.T.Ting 15g, *Bassia scoparia* (L.) A.J.Scott 20g, *Glycyrrhiza glabra* L. 6g. Modified based on different symptoms or signs.
Zhang 2006	Traditional Chinese Medicine Pediatrics[M]. 1997	Zhu Futang Practice of Pediatrics[M]. 1995	Removing dampness toxin, relieving fever and reducing fire	Modified Yinqiao powder, Wuwei Xiaodu decoction, Xijiao Dihaung decoction	*Bupleurum falcatum* L. 10-15g, *Ricinus communis* L. 10-15g, *Lonicera japonica* Thunb. 10-15g, *Strobilanthes cusia* (Nees) Kuntze 15-20g, *Taraxacum mongolicum* Hand.-Mazz. 10-15g, *Rehmannia glutinosa* (Gaertn.) DC. 10-15g, *Arnebia euchroma* (Royle ex Benth.) I.M.Johnst. 9-12g, *Dictamnus dasycarpus* Turcz. 10-15g, *Paeonia × suffruticosa Andrews 10-15g, Paeonia lactiflora* Pall. 10-15g, *Angelica dahurica* (Hoffm.) Benth. & Hook.f. ex Franch. & Sav. 6-12g, *Smilax glabra* Roxb. 10-15g, *Arctium lappa* L. 6-15g.
Yang 2003	Not report	Zhu Futang Practice of Pediatrics[M]. 1995	Clearing heat, removing toxin, draining dampness	Modified Yinqiao powder	*Forsythia suspensa* (Thunb.) Vahl 6-9g, *Lonicera japonica* Thunb. 6-9g, *Pterocarpus lucens* Lepr. ex Guill. & Perr. 6-9g, *Lophatherum gracile* Brongn. 6-9g, *Arctium lappa* L.3-6g, *Mentha canadensis* L. 3-5g, *Akebia trifoliata* (Thunb.) Koidz. 3-5g, *Glycyrrhiza glabra* L. 3-5g.
Li 2002	Traditional Chinese Medicine Pediatrics[M]. 1997	Not report	Clearing heat, removing toxin, removing dampness	Modified Wuwei Xiaodu decoction	*Lonicera japonica* Thunb 20g, *Chrysanthemum × morifolium* (Ramat.) Hemsl. 20g, *Rehmannia glutinosa* (Gaertn.) DC. 20g, *Strobilanthes cusia* (Nees) Kuntze 20g, *Isatis tinctoria* subsp. Tinctorial 20g, *Forsythia suspensa* (Thunb.) Vahl 10g, *Sophora flavescens* Aiton 15g, *Saposhnikovia divaricata* (Turcz. ex Ledeb.) Schischk. 15g.
Li 1999	Not report	Not report	Clearing heat, removing toxin, removing dampness, promoting diuresis	Modified Modified Yiyi Zhuye powder	*Coix lacryma-jobi* L. 15g, *Talcum* 15g, *Commelina communis* L. 15g, *Smilax glabra* Roxb. 12g, *Forsythia suspensa* (Thunb.) Vahl 9g, *Tetrapanax papyrifer* (Hook.) K.Koch 4g, *Scutellaria baicalensis* Georgi 6g, *Lophatherum gracile* Brongn. 6g, *Arnebia euchroma* (Royle ex Benth.) I.M.Johnst. 6g, *Arctium lappa* L. 6g, *Portulaca oleracea* L. 50g. Modified based on different symptoms or signs.
Wan 1996	The Standard for C M Diseases and Syn dromes therapeutic r esults[S]. 1995	Not report	Not report	Modified Qingwei powder	*Actaea cimicifuga* L., *Coptis chinensis* Franch., *Isatis tinctoria* subsp. tinctorial, *Lonicera japonica* Thunb., *Forsythia suspensa* (Thunb.) Vahl, *Gypsum*, *Rehmannia glutinosa* (Gaertn.) DC., *Arnebia euchroma* (Royle ex Benth.) I.M.Johnst. Biyu powder. Modified based on different symptoms or signs.
Lin 1994	Not report	Not report	Not report	Self-made prescription	Dipyridamole, *Botrychium ternatum* (Thunb.) Sw. 10-20g.
Ma 2017	Not report	Not report	Clearing heat and cooling blood, removing toxin and dampness	Shengma Gegen decoction, Baihu decoction, Xijiao Dihaung decoction	*Actaea cimicifuga* L. 10g, *Arnebia euchroma* (Royle ex Benth.) I.M.Johnst. 6g, *Cornu Bubali* 6g, *Paeonia × suffruticosa Andrews* 6g, *Spirodela polyrhiza* (L.) Schleid. 10g, *Arctium lappa* L 10g, *Pueraria montana var. lobata* (Willd.) Maesen & S.M.Almeida ex Sanjappa & Predeep 10g, *Anemarrhena asphodeloides* Bunge 10g, *Crataegus pinnatifida* Bunge 12g, *Cypsum* 15g, *Glycyrrhiza glabra* L. 6g.

eTable 3. Characteristics of included trials of herbal formulae for rubella

Study ID	Chinese medicine diagnostic criteria	Western medicine diagnostic criteria	Therapeutic principles and methods	Intervention	Herbal formulae
Li 2012	Not report	Infectious Diseases[M]. 2004	Dispersing wind, clearing heat, cooling blood	Modified Yinqiao powder, Xijiao Dihaung decoction	*Lonicera japonica* Thunb. 12g, *Forsythia suspensa* (Thunb.) Vahl 15g, 15g, *Mentha canadensis* L. 12g, *Bombyx Batryticatus*, *Nepeta cataria* L. 9g, *Lobelia chinensis* Lour. 15g, *Cyrtomium fortunei* J.Sm. 12g, *Paris polyphylla* Sm. 12g, *Pterocarpus lucens* Lepr. ex Guill. & Perr. 15g, *Oroxylum indicum* (L.) Kur 18g, *Glycyrrhiza glabra* L. 3g, *Paeonia × suffruticosa Andrew* 15g, *Rehmannia glutinosa* (Gaertn.) DC. 15g, *Arnebia euchroma* (Royle ex Benth.) I.M.Johnst. 15g.
He 2008	Not report	Rubella virus antibody positive	Reinforcing healthy qi and expelling pathogen, clearing heat and removing toxin	Huanglan Granule	*Astragalus mongholicus* Bunge 30g, *Strobilanthes cusia* (Nees) Kuntze 30g, *Cyrtomium fortunei* J.Sm. 30g.
Zhou 1995	Not report	Handbook of infectious diseases[M]. 1984	Dispersing wind and clearing heat, removing toxin and promoting eruption; Clearing heat and removing toxin, activating blood and cooling blood	Modified Yinqiao powder	*Lonicera japonica* Thunb., *Forsythia suspensa* (Thunb.) Vahl, *Mentha canadensis* L., *Lophatherum gracile* Brongn., *Arctium lappa* L., *Platycodon grandiflorus* (Jacq.) A.DC., *Glycyrrhiza glabra* L., *Pterocarpus lucens* Lepr. ex Guill. & Perr, *Glycyrrhiza glabra* L.. Modified based on different symptoms or signs.
